# The ten-year evaluation of clinical characteristics in congenital lung anomaly in pediatrics; a retrospective study in North of Iran

**DOI:** 10.1186/s12887-024-04911-y

**Published:** 2024-07-06

**Authors:** Narges Lashkarbolouk, Mahdi Mazandarani, Ali Ahani Azari, Somayeh Ghorbani, Lobat Shahkar

**Affiliations:** 1 Golestan University of Medical Sciences, Gorgan, Iran; 2 Endocrinology and Metabolism Research Center, Tehran University of Medical Sciences, Tehran, Iran; 3 Taleghani Pediatric Hospital, Golestan University of Medical Sciences, Gorgan, Iran

**Keywords:** Congenital lung anomaly, Congenital pulmonary airway malformations, Pulmonary hypoplasia, Bronchopulmonary sequestrations

## Abstract

**Introduction:**

Congenital lung anomalies (CLA) are a group of anomalies, including congenital cystic adenomatoid malformation (CCAM), bronchopulmonary sequestrations (BPS), congenital lobar emphysema (CLE), and bronchogenic cysts (BC). The prevalence of these rare anomalies has risen in recent years, according to various population-based studies due to advances in fetal ultrasound technology.

**Method:**

This retrospective study examines the diagnosis of CLA, and was conducted on 72 patients between March 2014 and March 2024 at Taleghani Pediatric Hospital in Gorgan, Iran.

**Result:**

The average age was 18.8 ± 30.3 months, with the majority being boys (62.5%). Most participants had CCAM (41.7%), followed by CLE (18.1%), BPS (16.7%), pulmonary hypoplasia (9.7%), BC (8.3%), and hybrid lesion (5.6%). The majority of patients were Fars (62.5%), and the average hospitalization days was 9.4 ± 4.5 days. Cardiac anomalies were observed in 19.4% of the patients. 62 patients (86.1%) exhibited respiratory symptoms, and prenatal screening during pregnancy led to the diagnosis in 51 patients (70.8%). Most patients had left lung anomalies (43; 59.7%), and the majority (90.3%) survived. There is a statistically significant relation between needed for surgical treatment and patients’ type of pulmonary lesions (*p*-value: 0.02). In addition, there was a significant relation between the Fars ethnicity and the presence of cardiac anomalies (*p*-value: 0.04).

**Conclusion:**

Some CLAs remain undiagnosed or untreated due to the rare nature of congenital lung anomalies. Nevertheless, improvements in ultrasound and other imaging methods will make diagnosing and managing these anomalies during the prenatal period more prevalent, resulting in enhanced understanding.

## Introduction

Lung lesions in children are primarily benign pulmonary anomalies with a wide range of clinical conditions and histopathology. The phrase “congenital lung anomaly” (CLA) encompasses a wide range of disorders, including congenital pulmonary airway malformation (CPAM or, previously known as congenital cystic adenomatoid malformation (CCAM)), bronchopulmonary sequestration (BPS), bronchogenic cysts (BC), congenital large hyperlucent lobe (CLHL), pulmonary hypoplasia (PH), and bronchial atresia [1]. The incidence has increased in recent years, according to several population-based studies, which suggest a frequency of 1 in 2,500 live births [2]. Moreover, Based on The European Surveillance of Congenital Anomalies (EUROCAT) data from 2008 to 2012, there was a prevalence of 1.05 cases of CCAM per 10,000 pregnancies, demonstrating a rising trend over four consecutive years [3]. According to Vatankhah S., et al., 2017, the general occurrence of congenital anomalies among infants in Iran is 2.3%, with respiratory anomalies having a prevalence of 1.82% [4]. This rising incidence is mainly due to the extensive availability of prenatal ultrasound along with the advanced resolution of ultrasound technology that allows the detection of smaller lesions in the respiratory system [1, 5].

Studies claim that up to 5% of children exhibit symptoms within the initial five years of life. However, a significant percentage (36–97%) of asymptomatic children at birth continue to remain symptom-free throughout their childhood. The majority of the patients present with respiratory distress, cardiovascular strain, mediastinal displacement, pneumothorax, and recurrent lower respiratory tract infections [1, 6, 7]. Additionally, in some cases, such as in CCAM lesions, patients carrying oncogenic driver mutations (e.g., KRAS) may pose an elevated risk for malignant transformation [8]. Histological characteristics of CLA have been linked to well-known pulmonary tumors, including rhabdomyosarcoma, pleuropulmonary blastoma, and bronchioalveolar carcinomas [1].

For confirming the diagnosis of CLA, the primary method is a postnatal chest CT within the first year of life. CLA frequently resolves spontaneously during fetal development and becomes undetectable during repeated prenatal ultrasound examinations. However, a chest CT scan after birth can identify nearly all lesions (98%) [1, 5, 9].

The antenatal prognosis is primarily associated with the size of the lesion. In a minority of cases (3–4%), intrauterine death or termination may occur. Following birth, a small percentage (1%) experience mortality due to respiratory failure during the neonatal period without undergoing surgery or after undergoing attempted emergency resection (7%). For those who survive the neonatal period, the long-term prognosis and outcome is influenced by various factors such as the development of symptoms, the need for or choice of surgical resection, the extent of resection, encountered pathology, and other underlying health conditions [1, 2, 5].

Children may encounter challenges in effectively communicating their symptoms, potentially leading to treatment in outpatient care as a consequence of lung complications arising from an underlying condition, like recurrent pulmonary infections. Such circumstances may contribute to delayed diagnosis, suboptimal treatment, and unfavorable results. The primary objective of our study was to illustrate the range of congenital pulmonary anomalies, as well as to provide a detailed report of demographic information, outcomes, and the varied clinical presentations exhibited by patients. The study’s secondary aims are to identify associated anomalies and their treatment choices. With the increasing incidence of pulmonary anomalies and the importance of prompt identification for suitable intervention and improved prognosis, this investigation delves into the ten-year prevalence of pulmonary anomalies at Taleghani Pediatric Hospital in Gorgan, Iran.

## Materials and methods

### Study design and participant selection

This retrospective study was conducted on CLA patients between March 2014 and March 2024 at Taleghani Pediatric Hospital in Gorgan, Iran. The study’s inclusion criteria encompass pediatric patients with confirmed CLA who have been referred to these centers. Exclusion criteria comprised pediatric patients without a confirmed diagnosis or with incomplete information.

### Data collection

Patient characteristics, including demographic and clinical data, were gathered through a patient record system. Data was collected based on a checklist of variables. The following data was retrospectively analyzed using medical records: gender, gestational age, surgery, patient’s initial symptom, length of hospital stay, lung involvement pattern, and accompanying features. For follow-up assessments, the patients underwent clinical evaluations conducted by a specialist in pediatric pulmonology at intervals of one month during the initial half-year period, at bi-monthly intervals during the subsequent six months, and at three-month intervals in the final six months. Then, based on patients’ condition the follow-up continues as needed. Additional diagnostic tests were prescribed as deemed necessary.

### Ethics approval and consent to participate

Informed consent was obtained from all legal guardians in the study. A copy of the written consent is available by the Editor of this journal. The purpose of this research was thoroughly explained to the legal guardians, and they were assured that the researcher would keep their information confidential. This study was conducted following the principles of the Declaration of Helsinki. The ethical committee of Golestan University of Medical Sciences approved the study. Ethical code: IR.GOUMS.REC.1402.237.

### Statistical analysis

Data was analyzed using Statistical Package for Social Sciences version 25.0 (SPSS Inc, Chicago, IL). Chi-square and Student’s t-test were employed to test and compare the relationship between variables. A *P*-value less than 0.05 is deemed statistically significant. The chi-square test was utilized to compare the ratio of qualitative variables among the groups if the presuppositions of chi-square distribution were established. Otherwise, Fisher’s exact test was used. The results are expressed as mean ± standard deviation or standard error.

## Result

In our study, 72 patients were included between 2014 and 2024 after applying the inclusion and exclusion criteria. Table 1 shows the Distribution and Characteristics of Congenital Pulmonary Anomalies. The average age of our patients was 18.8 ± 30.3 months, with the majority being boys (45 cases; 62.5%). Most participants had CCAM (30; 41.7%), followed by CLE (congenital lobar emphysema) (13; 18.1%), BPS (12; 16.7%), PH (7; 9.7%), BC (6; 8.3%), and HL (hybrid lesion) (4; 5.6%). The majority of patients were Fars (45; 62.5%), while 17 (23.6%) patients were Turkmen, and 10 (13.9%) patients were Sistani. The average hospitalization days for patients was 9.4 ± 4.5 days.

**Table 1:** Distribution, frequency, and characteristics of congenital pulmonary anomalies

Variables		Frequency	Percent
Gender	Boy	45	62.5%
	Girl	27	37.5%
Ethnicity	Fars	45	62.5%
	Turkman	17	23.6%
	Sistani	10	13.9%
Diagnosis	CCAM	30	41.7%
	CLE	13	18.1%
	BPS	12	16.7%
	PH	7	9.7%
	BC	6	8.3%
	HL^†^	4	5.6%
Lung involvement	Left side	43	59.7%
	Right side	23	31.9%
	Both sides	6	8.3%
Respiratory manifestation	Yes	62	86.1%
	No	10	13.9%
prenatal diagnosis	Yes	51	70.8%
	No	21	29.2%
Surgical Treatment	Yes	12	16.7%
	No	60	83.3%
Cardiac anomalies	Yes	14	19.4%
	No	58	80.6%
Other anomalies	Yes	7	9.7%
	No	65	90.3%
Outcome	Survive	65	90.3%
	Mortality	7	9.7%

CCAM: congenital cystic adenomatoid malformation, CLE: congenital lobar emphysema, BPS: bronchopulmonary sequestration, BC: bronchogenic cysts, HL: hybrid lesion, PH: pulmonary hypoplasia

^†^ Hybrid lesion defined as concurrent presence of CCAM and sequestration in an individual pulmonary lesion

Cardiac anomalies along were observed in approximately 19.4% (14 cases) of the patients. In 9.7% of patients, other anomalies such as craniofacial, limb, and gastrointestinal abnormalities were seen. In our cases, we observed intestinal malrotation and bronchoesophageal fistula.

Before diagnosis, 62 patients (86.1%) exhibited respiratory symptoms; prenatal screening during pregnancy led to the diagnosis in 51 patients (70.8%), and the mean gestational age for diagnosis was 37.9 ± 1.1 weeks. The majority of patients had left lung anomalies (43; 59.7%), followed by right lung anomalies (23; 31.9%), and both lung anomalies (6; 8.3%). Twelve patients underwent surgical treatment. Most patients (90.3%) survived, and only seven (9.7%) died. All patients (12 patients) underwent surgical treatment for symptoms of the congenital lung anomaly and associated cardiac and craniofacial anomalies. Two patients (BPS and PH) died due to surgical complications. Five patients died from complications due to accompanying anomalies, and the result of sepsis, acute respiratory distress syndrome (ARDS), and necrotizing enterocolitis (NEC). Three of them died at the age of neonates (two patients had craniofacial and gastrointestinal abnormalities, and one patient had cardiac anomalies). Two of them died at the age of infant (one patient had craniofacial and limb abnormalities, and one patient had cardiac anomalies).

Table 2 demonstrates the general characteristics of the patients included in our study according to pulmonary lesion diagnosis. There is a statistically significant relation between needed for surgical treatment and patients’ type of pulmonary lesions (*p*-value: 0.02). However, there was no significant relation between the kind of pulmonary lesions and other variables in the study.

**Table 2:** General characteristics of included patients according to pulmonary lesion diagnosis

Variables		Diagnosis				***P***-value
		CLE	CCAM	BPS	Others^*^	
Ethnicity	Fars	8	22	5	10	0.28
	Others	5	8	7	7	
Gender	Boy	9	20	7	9	0.74
	Girl	4	10	5	8	
Surgical treatment	Yes	1	3	1	7	0.02
	No	12	27	11	10	
Cardiac Anomalies	Yes	2	8	1	3	0.56
	No	11	22	11	14	
Other Anomalies	Yes	0	5	1	1	0.33
	No	13	25	11	16	
Prenatal Diagnosis	Yes	9	19	10	13	0.57
	No	4	11	2	4	
Outcome	Survive	11	30	10	14	0.13
	Mortality	2	0	2	3	
Lung involvement	Left Side	9	16	8	10	0.64
	Right Side	2	12	4	5	
	Both Side	2	2	0	2	
Respiratory Manifestation	Yes	11	28	9	14	0.42
	No	2	2	3	3	

CCAM: congenital cystic adenomatoid malformation, CLE: congenital lobar emphysema, BPS: bronchopulmonary sequestration

^*^Others include bronchogenic cysts (BC), hybrid lesions (HL), and pulmonary hypoplasia (PH).

In addition, there was a significant relation between the Fars ethnicity and the presence of cardiac anomalies in the patients (*p*-value: 0.04). Moreover, although there was no significant relation between gender and the presence of respiratory symptoms, it was statistically close to being significant (*p*-value: 0.05). There is also a significant relation between types of CLA and needed for surgical treatment. Patients with BC, HL, and PH had more surgical treatment (*p*-value: 0.02).

Follow-up appointments and imaging studies for at least 18 months for all patients have done to find out improvement the patients’ conditions and treatment intervention. Nonetheless, until now, regular monitoring and follow-ups with healthcare providers have been done to ensure that no changes or complications arise over time.

## Discussion

The incidence of pulmonary lesions has recently risen due to improved prenatal screening scans during the mid-trimester [10, 11]. These lesions, which can manifest as cystic and/or solid lesions, may either grow progressively, leading to a shift in the mediastinal area or regress partially or entirely before birth. Antenatal complications of CLA include conditions such as fetal hydrops, pleural effusion, or polyhydramnios [1, 2, 12]. Similar to our study, 70.8% of patients were diagnosed through prenatal screening. As stated in the studies, the most common types of CLA include CCAM, BPS, BC, and CLE [1, 2]. In our study, most of our cases were diagnosed with CCAM (41.7%), CLE (18.1%), BPS (16.7%), BC (8.3%), and HL (5.6%).

In our investigation, it was observed that pulmonary lesions exhibit a higher incidence among boys compared to girls, with an average hospitalization period of 9.4 ± 4.5 days. This aligns with the findings in Shahkar L, et al. 2016, research, which explored 47 cases of individuals with pulmonary lesions, similarly noting a male preponderance (28 cases; 75%) and an average hospitalization stay of 13.3 ± 10.2 days. Moreover, their study highlighted a substantial proportion of patients diagnosed with CLE (43.5%) and CCAM (32.5%), a trend that mirrors our research outcomes [13]. However, in the study conducted by Langston C., et al., 2003, they observed that BPS (40%), CLE (24%), CCAM (21%), and BC (15%) were most frequent among their 42 cases [14].

There is a disparity in reproductive outcomes across various racial and ethnic groups, with African Americans facing a higher risk of preterm delivery, low birth weight, and infant mortality compared to Caucasians. Research has highlighted differences in the prevalence of specific birth defects among different racial and ethnic populations. Egbe A., et al., 2015, found that the overall risk of congenital anomalies was comparable between Caucasians and Asians, but Asians had a heightened risk of craniofacial and musculoskeletal anomalies [15]. Our study identified a significant association between Fars ethnicity and the occurrence of cardiac anomalies in patients (*p*-value: 0.04). Nevertheless, there was no significant relation between other anomalies (e.g. craniofacial anomalies) and variables in the study [16].

Normally, the delivery of these patients is uncomplicated; however, the clinical presentation of CLA varies after birth. More than 75% of newborns do not display symptoms, with only a small number requiring respiratory support [1, 17]. According to the studies, patients are mostly symptomatic around seven months of age. Symptoms are often associated with infections, chronic cough, or recurrent wheezing, although most infants remain asymptomatic [1, 18, 19]. Most of our cases presented with respiratory manifestations at the time of diagnosis (86.1%).

If CLA is left undiagnosed and untreated in patients, complications such as bacterial, fungal, and mycobacterial infections, bleeding leading to hemothorax, high-output cardiac failure, pneumothorax, air embolism, and potentially malignant conditions can be expected. These conditions seem to be found in 3.2% of untreated patients [1, 20].

Pulmonary hypoplasia (PH) is a congenital defect characterized by stunted growth and development of lung tissue, airways, and blood vessels, with an estimated incidence of nine to eleven cases per 10,000 births. In addition, studies based on perinatal autopsy data have shown that the prevalence of PH ranges from 7.8 to 26%, which makes it the most commonly identified anomaly in cases of perinatal mortality. The symptoms vary depending on how much the lungs are affected, ranging from severe bilateral cases to less severe unilateral or lobar cases. In newborns, symptoms may include pulmonary hypertension, or respiratory insufficiency [21, 22]. In our research, seven patients were diagnosed with PH. The patients ranged in age from 1 month to 3 years old. All of them presented with symptoms of respiratory distress and were found to have underdeveloped lungs upon further investigation.

It should be noted that during our investigation, we found that patients with undiagnosed congenital anomalies or other pulmonary lesions who presented with pulmonary symptoms were treated on an outpatient basis with the diagnosis of pulmonary infection [23–26]. Because children’s respiratory systems are still developing and their immune systems may not effectively address defects, coupled with the high risk and infrequent nature of surgery in children, as well as the absence of clear treatment guidelines for these patients, it is crucial to ensure timely diagnosis in such cases.

The strengths of our study include a ten-year review of all referral cases, follow-up, and prenatal information of CLA patients. Limitations of our study include a retrospective study with potential bias in reliance on medical records. Additionally, more studies are necessary to discover factors that could diagnose pulmonary abnormalities sooner and with less invasive techniques. Furthermore, patients requiring surgical intervention were transfered to the advanced centers as our hospital lacked the necessary equipment for invasive procedures.

### Conclusion

Even though the majority of innate lung abnormalities are typically identified early in a person’s life, some patients may only exhibit symptoms later on. It is important to thoroughly assess the associated anomalies including cardiovascular systems during the initial evaluation of newborns. In addition, it is recommended that parents of patients be provided with genetic counselling services. By using advanced diagnostic methods and thorough physical evaluations, timely recognition and suitable management of these lesions can be accomplished.

## Data Availability

The data supporting this study’s findings are available from the corresponding author upon reasonable request.
